# Epigenetic Regulation of p21^cip1/waf1^ in Human Cancer

**DOI:** 10.3390/cancers11091343

**Published:** 2019-09-11

**Authors:** Matthias Ocker, Samar Al Bitar, Ana Carolina Monteiro, Hala Gali-Muhtasib, Regine Schneider-Stock

**Affiliations:** 1 Bayer AG, Translational Medicine Oncology, 13353 Berlin, Germany; 2 Department of Gastroenterology, CBF, Charité University Medicine Berlin, 10117 Berlin, Germany; 3 Department of Biology, American University of Beirut, Beirut 110236, Lebanon; 4 Experimental Tumor Pathology, Institute of Pathology, University Hospital, Friedrich-Alexander University Erlangen-Nuremberg, 91054 Erlangen, Germany; 5 Center for Drug Discovery, American University of Beirut, Beirut 110236, Lebanon; 6 Experimental Tumor Pathology, FAU Erlangen-Nuremberg, Universitaetsstrasse 22, 91054 Erlangen, Germany

**Keywords:** p21^cip1/waf1^, DNA methylation, histone acetylation, histone methylation, lncRNA, miRNA, epigenetic reader, epigenetic writer, epigenetic eraser

## Abstract

p21^cip1/waf1^ is a central regulator of cell cycle control and survival. While mutations are rare, it is commonly dysregulated in several human cancers due to epigenetic mechanisms influencing its transcriptional control. These mechanisms include promoter hypermethylation as well as additional pathways such as histone acetylation or methylation. The epigenetic regulators include writers, such as DNA methyltransferases (DNMTs); histone acetyltransferases (HATs) and histone lysine methyltransferases; erasers, such as histone deacetylases (HDACs); histone lysine demethylases [e.g., the Lysine Demethylase (KDM) family]; DNA hydroxylases; readers, such as the methyl-CpG-binding proteins (MBPs); and bromodomain-containing proteins, including the bromo- and extraterminal domain (BET) family. We further discuss the roles that long noncoding RNAs (lncRNAs) and microRNAs (miRNAs) play in the epigenetic control of p21^cip1/waf1^ expression and its function in human cancers.

## 1. Introduction

Regulation of gene function is essential to the coordination of cellular processes and tissue homeostasis. In cancer, dysregulation of genes is commonly mediated by genetic events such as activating mutations (e.g., *k-RAS, PI3K, EGFR*), gene fusions (e.g., *BCR-ABL*, *NTREK*), or amplifications (e.g., *FGFR*). Besides these mechanisms, additional features “on top” of the genetic information, i.e., epigenetic modifications, are heavily involved in the orchestration of gene functions. The term ‘epigenetics’ was first used by Conrad Hal Waddington in 1942 and is now defined as inherited changes in gene expression without alterations in the DNA [1,2]. While Hanahan and Weinberg defined the hallmarks of cancer based largely on genetic alterations, an increasing body of evidence now supports the view that epigenetic mechanisms contribute to the development of the malignant phenotype and can even drive cancer formation on their own [3,4].

An epigenetic signature is mediated by enzymes that alter DNA methylation or modifications of histones via methylation, acetylation, phosphorylation, ubiquitination, and various other mechanisms [5]. Besides these so-called epigenetic writers and erasers, enzymes that “read” the chromatin state form part of the epigenetic machinery [6]. Furthermore, various noncoding RNAs have recently been identified as playing a major role in epigenetic regulation [7].

p21^cip1/waf1^ is a low-molecular-weight molecule (21 kDa) that inhibits cyclin-dependent kinases. It is a key downstream target of p53 upon DNA damage and has multiple functions during cell cycle regulation, DNA repair, gene transcription, and apoptosis [8]. We have previously discussed the link between histone deacetylase inhibitors (HDACi) and p21^cip1/waf1^ signaling [9]. Here, we will extend this discussion to epigenetic mechanisms, such as DNA or histone methylation, and their effects on p21^caip1/waf1^ in cancer cells.

## 2. Epigenetic Mechanisms

### 2.1. Epigenetic Writers

The epigenetic landscape of tumor cells is created by various chemical modifications at DNA or histone tails.

DNA methylation occurs in somatic cells at the so-called CpG (cytosine–phosphate–guanine) islands, mostly CG-rich DNA sequences in the gene promoter regions and usually leads to silencing of the respective gene. This modification is mediated by the DNA methyltransferase (DNMT) family [10]. In cancer, about 5–10% of promoter regions become abnormally methylated [11]. DNMT1 is considered to be a maintenance DNA methyltransferase that copies methylation patterns during DNA replication and is thus important to the inheritance of DNA methylation, while DNMT3a and DNMT3b act as de novo DNA methyltransferases during embryogenesis and in somatic tissues [12,13]. Although the promoter region of *CDKN1A*, the gene encoding p21^cip1/waf1^, is usually not methylated in acute myeloid leukemia (AML), decitabine is capable of inducing its re-expression via induction of DNA damage and activation of the ATM/p53 pathway [14].

Histone modifications make a major contribution to the transcriptional outcome of a specific gene sequence. Interestingly, a histone lysine residue can be modified differentially; i.e., it can be acetylated or (tri-)methylated, and each modification will contribute to cancer development in a different way and extends the language of the DNA sequence [15,16]. Lysine acetyltransferases (KATs) catalyze the transfer of an acetyl group from acetyl–CoA to the ε-amino group of a lysine. Histone acetylation is a modification that can be recognized by epigenetic readers, but it also leads to a more relaxed chromatin structure and, consequently, an enhanced binding of transcription factors. Acetylation can occur at different histone tail lysines (K9, K14, K18, K5, K8, and K12) and in non-histone proteins. TIP60 and GCN5 are further acetyltransferases that link epigenetic modifications to p21^cip1/waf1^, e.g., by acetylation of p53 and MYC, respectively [17,18,19].

H3K4 methylation in general marks a transcriptionally active gene. There are six methyltransferases (MLL1/KMT2A, MLL2/KMT2B, MLL3/KMT2C, MLL4/KMT2D, SETD1A/KMT2F, and SETD1B/KMT2G) that lead in different complexes to this active histone modification. Mutations and rearrangements of the histone-lysine N-methyltransferase 2 (MLL) family proteins play a major role in the initiation and maintenance of various cancer types [20]; p53 might be involved in the correct recruitment of the Set1 complex to DNA-damage-responsive genes and thus links this phenotype to p21^cip1/waf1^ [21]. The association of methyltransferases in diseases has recently been summarized in Hyun et al. [22].

The H3K27me3 mark is associated with gene silencing. The responsible Polycomb repressive complex 2 (PRC2) is composed of four core subunits: EZH2, SUZ12, EED, and RbAp46/48. The catalytic subunit EZH2 is responsible for the mono-, di-, and trimethylation of lysine 27 on histone H3 [23]. EZH2 is upregulated in many cancers, and the inhibition of PRC2 catalytic activity has emerged as a promising therapeutic approach in cancer [24,25].

There are at least eight different H3K36 methyltransferases; the most important are NSD1/KMT3B, NSD2/KMT3G, NSD3/KMT3F, and SETD2/KMT3A [26].

Dot1L is responsible for the active mark at H3K79 that is localized at the globular domain of histone 3. The relationship of the abovementioned epigenetic writers to p21^cip1/waf1^ is summarized in Table 1 and discussed in further detail below.

### 2.2. Epigenetic Erasers

Modifications to DNA and histones need to be reversible to be functional regulators of gene activity and are performed by distinct enzymes that are classified as epigenetic erasers. Generally, the removal of methylation marks facilitates transcription and enhances gene expression, while deacetylation has suppressive effects.

DNA methylation can be reversed by the ten-eleven translocation (TET1-3) family of proteins [27]. TET proteins are DNA hydroxylases that convert 5-methylcytosine to 5-hydroxymethylcytosine and, if the oxidization process is repeated, to 5-formylcytosine and 5-carboxylcytosine [28,29,30]. 5-hydroxymethylcytosine is commonly found in sites of active chromatin, such as promoters and transcriptional start sites, and it has been identified as a recognition signal for epigenetic readers [31,32]. Mutations in TET2 (leading to decreased 5-hydroxymethylcytosine levels) have been associated with various hematologic malignancies [33], although the exact mechanism of how TET2 contributes to the development of such cancers remains unclear [34]. Epigenetic inactivation or reduced expression of TET3 has been demonstrated in solid tumors such as head and neck, cervical, or colon cancer [35,36,37].

Histone lysine demethylases (KDMs) consist of more than 30 enzymes that have a distinct substrate specificity and belong to two different enzyme families: the Flavin-Adenin-Dinukleotid (FAD)-dependent amine oxidase family with KDM1A and KDM1B (LSD1, LSD2) as the only two members and the Jumonji C-domain-containing family of dioxygenases with more than 30 members in several subfamilies (KDM2–8). KDM1A/B can remove mono- and dimethyl groups from histone H3K4, while the Jumonji-family enzymes can also remove trimethyl groups [38,39]. So far, no clear evidence for specific histone arginine demethylases exists. Peptidylarginine deiminase 4 (PAD4) and JMJD6 have been shown to mediate arginine demethylation, but both enzymes also possess additional properties beyond demethylation [40,41].

Histone deacetylation is removed by zinc-dependent Class I, II, and IV histone deacetylases (HDAC) or Nicotinamide adenine dinucleotide + (NAD+)-dependent sirtuins that are also referred to as Class III HDACs. HDACs remove ε-amino acetyl groups from lysine residues in histones and in non-histone proteins (especially mediated by Class II HDACs, which are also found in the cytoplasm), which explains the pleiotropic effects and rather narrow therapeutic window of HDAC inhibitors (HDACi) in humans [42,43]. HDACs are part of larger multi-enzyme complexes, including Sin3, NuRD, and Co-REST, that determine their target recognition and substrate specificity, but also have demethylase properties [44,45]. Mechanistically, removal of acetylation from histones restores a condensed and transcriptionally repressive status. While mutations in HDACs are uncommon, altered expression levels of HDACs have been described in various human cancers and proposed as prognostic markers, e.g., in colorectal, ovarian, breast, and liver cancer [46,47,48,49].

### 2.3. Epigenetic Readers

Epigenetic modifications are recognized by a group of proteins that are referred to as epigenetic readers. Most reader proteins specifically recognize and interact with specific epigenetic marks; however, some readers also bind to unmodified histones whose functional relevance is not well understood yet [50,51,52,53].

Methylated CpG islands in DNA are recognized by methyl-CpG-binding proteins (MBPs), and these proteins in general support the gene silencing properties of methylated loci. Three families of MBPs have been described: MBD proteins (MBD1-6, SETDB1/2, BAZ2A/B, and MeCP2), Zinc Finger (ZnF) domain proteins (ZBTB4, ZBTB33/Kaiso, ZBTB38, and ZFP57), and the SET and RING finger-associated (SRA) domain proteins (UHRF1) [6,54].

Histone lysine methylation marks are recognized by a plethora of different protein families that specifically interact with distinct lysine residues and their methylation pattern [55,56]. Interestingly, a cross-talk between DNA and histone methylation exists by, e.g., interaction of the ATRX, DNMT3, DNMT3L (ADD) domain of DNMT3 with the histone H3 or the binding of the DNA methylation reader UHRF1 with unmethylated H3R2 [57,58,59].

Acetylation of histones leads to an open chromatin structure that is recognized by proteins containing bromodomains or tandem plant homeodomain (PHD) domains [60,61]. In humans, more than 60 bromodomains have been identified [62]. Among them, the bromo- and extraterminal domain (BET) family proteins (BRD2, BRD3, BRD4, and BRDT) are the best studied and have been shown to regulate transcriptional elongation processes as well as cell cycle progression. Interestingly, inhibition of BET proteins leads to downregulation of MYC, which is a key regulator of cellular survival and proliferation (e.g., via ^p21cip1/^waf1) in hematologic and solid tumors [63,64,65]. Other acetylation readers, such as EP300 and CREBBP, possess only a single bromodomain and are also known to regulate gene transcription processes [66].

## 3. Epigenetic Regulation of p21^cip1/waf1^

### 3.1. Promoter Methylation of CDKN1A

Expression of p21^cip1/waf1^ is regulated by several epigenetic and other additional post-transcriptional mechanisms that are also interconnected with each other (Tables 1 and 2).

The proximal promoter of *CDKN1A* contains several CpG dinucleotides that can be subject to DNA methylation [67,68]. Methylation affects Sp1 and Sp3 binding activity and was shown to suppress p21^cip1/waf1^ transcription independently of p53 [69,70]. Some anticancer compounds, such as curcumin, induce apoptosis by promoter demethylation of *CDKN1A* [68]. In prolactinoma, low p21^cip1/waf1^ levels caused by *CDKN1A* promoter hypermethylation were shown to contribute to enhanced proliferation in invasive specimens [71].

Hypermethylation of the Sp1 and Sp3 genomic region and concomitant transcriptional repression were found in different hematologic and solid cancers, including breast cancer, and were associated with a poorer prognosis due to more aggressive phenotypes [72,73,74]. DNMTs, as mediators of DNA methylation, have thus also been shown to have elevated levels in cancer, and pharmacological inhibition of these enzymes is already a clinical standard in hematologic diseases [75]. Furthermore, DNMTs compete with p21^cip1/waf1^ for PCNA binding and overexpression of DNMTs could thus release p21^cip1/waf1^ from PCNA and render it more susceptible to proteasomal degradation [76,77].

Nevertheless, low p21^cip1/waf1^ levels in tissues are not always associated with *CDKN1A* promoter hypermethylation as previously shown in osteoarthritic chondrocytes [78].

**Figure 1:**
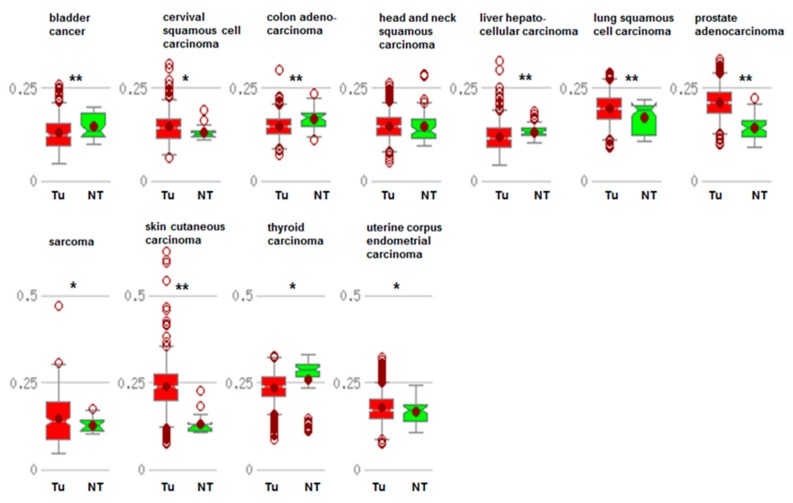
Comparison of average *CDKN1A* gene expression in a tumor sample (Tu) and matched normal samples (NT) in a panel of different tumor types. Data were taken from the MethHC database (http://methhc.mbc.nctu.edu.tw/) for p21^cip1/waf1^ (NM-000389). * *p* < 0.05; ** *p* < 0.005.

**Figure 2:**
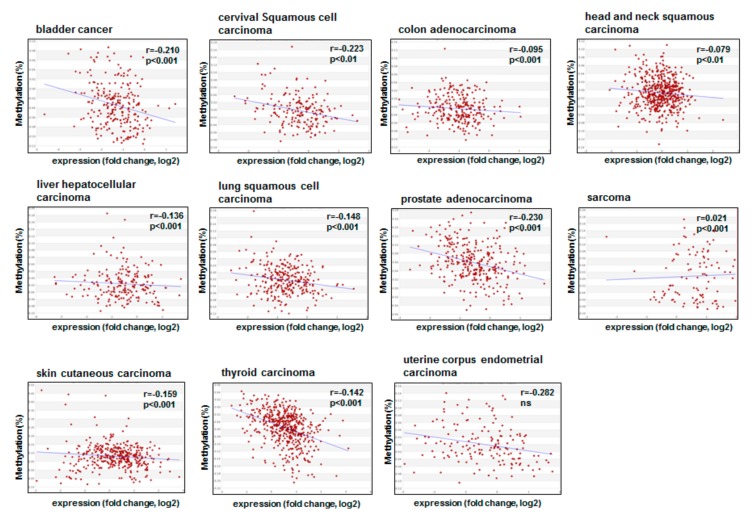
Comparison of promoter DNA methylation level and mRNA expression in tumor samples in a panel of different tumor types. ß values for methylation are between 0 and 1: 0—unmethylated and 1—fully methylated. Data were taken from the MethHC database (http://methhc.mbc.nctu.edu.tw/) for p21^cip1/waf1^(Nm-000389). *r*, correlation coefficient.

Analyzing in silico the MethHC database that integrates DNA methylation and mRNA expression data from the Cancer Genome Atlas (TCGA), we observed that there is no uniform pattern when comparing tumor with nontumor DNA for *CDKN1A* gene expression. Bladder cancer, colon cancer, liver hepatocellular carcinoma, and thyroid carcinoma show a lower *CDKN1A* expression in tumors, whereas cervical squamous cell carcinoma, lung squamous carcinoma, prostate adenocarcinoma, sarcoma, skin cutaneous carcinoma, and uterine corpus endometrial carcinoma show a higher p21^cip1/waf1^ encoding mRNA expression in tumors compared to their normal counterparts (Figure 1). Head and neck carcinoma did not even show different gene expression levels. Except for prostate and skin carcinoma, there were no impressive differences visible to the naked eye in the graphs. As expected, when analyzing DNA promoter methylation and *CDKN1A* gene expression, there was a negative correlation between both parameters except for uterine corpus endometrial carcinoma; however, the correlation coefficients were not remarkably high (Figure 2). These results point to the fact that *CDKN1A* silencing is not epigenetically regulated by DNA methylation only. We summarize the major mechanisms in the following paragraphs.

### 3.2. Writers and p21^cip1/waf1^

Several histone acetyl transferases (HATs), e.g., p300, CREB-binding protein, PCAF, Tip60, and GCN5, have been shown to be crucial for p53-dependent and independent activation of p21^cip1/waf1^ [79,80] (Table 1). Interestingly, the histone acetyltransferase PCAF can induce the acetylation of H3K9 and H3K14 at the distal p53-responsive element in the *CDKN1A* promoter to induce its expression under stress conditions. This action is independent of PCAF-mediated acetylation at K320 in p53’s regulatory domain [79,80]. The TIP60 protein is a lysine acetyltransferase that can directly acetylate histones or non-histone proteins to alter their activity. TIP60 was shown to act as a tumor suppressor in vivo. In human papilloma virus (HPV)-mediated cervical cancer, TIP60 is destabilized by the HPV-mediated complex with the E3 ubiquitin ligase EDD1 [81]. High TIP60 expression was associated with 5-Fluoruracil (5-FU)-induced apoptosis and growth arrest and is not restricted to p53-dependent promoters [18]. The reason for this is that acetylation at the two TIP60-mediated acetylation sites Lys161 and Lys163 at the p21^cip1/waf1^ C-terminus leads to stabilization of p21^cip1/waf1^ and this acetylation is a pre-requisite for p21^cip1/waf1^-dependent DNA damage response and cell cycle progression.

SETD1A a histone methyltransferase that ensures that the active H3K4 methylation triggers cell proliferation by suppressing p21^cip1/waf1^ in a p53-independent manner. On the other hand, SETD1A knockdown leads to a p21^cip1/waf1^-dependent senescence phenotype [93]. The fact that SETD1A is often amplified in invasive breast carcinomas might reflect the better tolerance of these aggressive tumor cells to mitotic stress. Cell cycle arrest in SETD1A knockdown senescent cells is independent of mutations in p53, Rb, and p16, which are known senescence mediators; instead, it is sustained through transcriptional suppression of SKP2, which degrades p27 and p21^cip1/waf1^. The histone methyltransferase SETD2, which is responsible for the trimethylation of H3K36, is necessary for the proper mismatch repair in vivo [35]. In this regard, it has been shown that the hMutSα subunit of the mismatch repair protein hMSH6 contains a Pro–Trp–Trp–Pro (PWWP) domain that reads the H3K36me3 histone mark [94]. SETD2 can interact with p53 to regulate the transcription of its target genes encoding for e.g. p21^cip1/waf1^, Fas, and Puma [95]. SETD2 loss could also promote tumor growth in a p53-independent manner, leading to aberrant mitosis and multinucleated giant cells in tumors.

In lymphoma, the growth factor independent 1 (Gfi1) oncoprotein mediates the recruitment of the histone lysine methyltransferase G9a in complex with the histone deacetylase HDAC1 to the *CDKN1A* promoter. The subsequent increase in H3K9 dimethylation leads to diminished *CDKN1A* transcription [36]. Recently, the COUP-TF-interacting protein 2 (CTIP2) and the histone methyltransferase SUV39H1 were shown to be recruited to the proximal SP1 site of the *CDKN1A* promoter, and trimethylation of H3K9 was found to lead to transcriptional repression of *CDKN1A* in microglial cells. In parallel, human immunodeficiency virus (HIV-1) infection of macrophages activates *CDKN1A* gene expression and promotes G2/M cell cycle arrest. Both actions seem to facilitate HIV-1 replication [37].

Overexpressed MYC induces acute erythroleukemia, an uncommon AML subtype that is associated with a worse prognosis. The histone methyltransferase EZH2 has been reported to be a target of the MYC oncoprotein. MYC knockdown results in reduced EZH2 expression and an increase in p21^cip1/waf1^ expression by removal of the silencing EZH2-specific H3K27me3 mark at the *CDKN1A* promoter. The subsequently induced p21^cip1/waf1^-dependent senescence phenotype is p53-independent [38]. In non-small-cell lung cancer, the long noncoding RNA LINCO1133 recruits EZH2 and lysine-specific demethylase 1A (LSD1) to the *CDKN1A* promoter to repress its transcription, leading to a proliferative stimulus [39]. Whereas EZH2 sets the silencing H3K27me3 mark, LSD1 removes the active H3K4me1/2 code to shape the chromatin into a repressive conformation. This p21^cip1/waf1^ suppression has also been associated with epithelial-to-mesenchymal transition, a process that is important for invasion and metastasis. Downregulation of methylation of H3K79 leads to a p21^cip1/waf1^-dependent induction of the senescence program in lung cancer, which is believed to represent a barrier to tumorigenesis. When tumor cells are deficient in the responsible methyltransferase DOT1, they show a remarkable chromosomal instability [88]. When investigating a panel of histone methyltransferases in prostate cancer, PRMT6, a Type I arginine methyltransferase that builds the H3R2 dimethylation, was highly upregulated compared to nontumor tissue [89]. PRMT6 counteracts the MLL complex at H3K4 and both marks are mutually exclusive. When the repressive H3R2me2 mark is lost, tumor aggressiveness is significantly reduced by inhibition of the oncogenic AKT pathway and senescence induction via p21^cip1/waf1^ upregulation. In leukemia, methylation at H4K20 by suppressor of variegation 4-20 homolog 1 (Suv4-20h1) promotes cell cycle G1/S transition and, hence, proliferation by repressing the p21^cip1/waf1^ encoding gene [90]. A direct binding of Suv4-20h1 with enrichment of histones H3K20me2 and H4K20me3 at the *CDKN1A* promoter was shown in that study.

The histone variant H2A.Z is enriched at p53 binding sites in the *CDKN1A* promoter, facilitating p53 binding and p21^cip1/waf1^ expression [91]. Interestingly, H2A.Z is lost from the *CDKN1A* promoter in response to DNA damage followed by recruitment of TIP60 histone acetyltransferase to trigger *CDKN1A* gene expression [92]. Thus, H2A.Z seems to be a negative regulator of p21^cip1/waf1^. The authors show that p53 plays an active role in positioning H2A.Z to gene promoters and contributes to p21^cip1/waf1^ induction in an H2A.Z-depleted setting. In contrast, MYC represses *CDKN1A* transcription by preferentially recruiting H2A.Z at MYC binding sites in the TATA initiator region of the *CDKN1A* promoter to protect the malignant cells from senescence [91,92].

These findings indicate a tight and complex crosstalk between different epigenetic mechanisms in regulating p21^cip1/waf1^ expression in different human cancer cells.

### 3.3. Erasers and p21^cip1/waf1^

Besides DNA methylation, histone H3 lysine methylation/demethylation was demonstrated to regulate p21^cip1/waf1^ expression (Table 2). Lysine-specific demethylase (LSD1/KDM1A) is a key regulator of histone H3K4 and H3K9 mono- and dimethylation [96]. It is commonly overexpressed and correlated with a poor prognosis in different human cancers [97,98,99]. LSD1 regulates p21^cip1/waf1^ [100] via demethylation of histone and non-histone targets, including p53 and DNMT1 [101], and controls the cell cycle via demethylation of Rb, interaction with long noncoding RNAs (lncRNAs) (including HOTAIR), and demethylation of AGO2, a key element of the RNA-induced silencing complex [96,102]. In glioma and renal tumor cells, LSD1 knockdown enhanced *CDKN1A* transcription. This was correlated with increased levels of H3K4me2 at the *CDKN1A* promotor [100,101] (Table 2). Surprisingly, in prostate cancer, LSD1 knockdown resulted in increased H3K4me2 in the *CDKN1A* promotor, but repressed *CDKN1A* transcription. However, LSD1 knockdown increased DNMT1 binding to the promotor; thus, methylation might be responsible for the reduced levels of p21^cip1/waf1^ [103].

Mutations of isocitrate dehydrogenase (IDH) lead to the generation of 2-hydroxoglutarate (2-HG), an oncometabolite, instead of the physiologic metabolite α-ketoglutarate (α-KG), which is a co-substrate for TET and Jumonji enzymes. 2-HG acts as a competitive inhibitor to these epigenetic modifiers and, thus, increases histone lysine methylation and decreases 5-hydroxymethylcytosine levels [107], which can lead to enhanced expression of p21^cip1/waf1^ [104]. Interestingly, IDH mutations are frequently observed in glioblastoma, intrahepatic cholangiocellular carcinoma, and acute myeloid leukemia, where epigenetic alterations are commonly found, and are already part of established diagnostic and therapeutic algorithms [108,109,110].

It is known that vitamin C can increase the levels of TET enzymes [111,112]. In skin cancer cells, this treatment induced a global DNA demethylation, which was associated with elevated 5hmC levels in the *CDKN1A* promotor and increased p21^cip1/waf1^ expression. Knockout of the enzymes TET1/TET2 inhibited p21^cip1/waf1^ upregulation, suggesting that TET regulates p21^cip1/waf1^ expression in skin cancer cells treated with vitamin C [113].

In agreement with our previous report [9], recent studies have continued to demonstrate the regulation of p21^cip1/waf1^ expression by HDAC inhibitors (HDACi) in tumors. In non-small-cell lung carcinoma, treatment with HDACi alone or in combination with the natural flavonolignan silibinin caused increased H3 and H4 acetylation in the Sp1/Sp3 binding site at the *CDKN1A* promoter, activating *CDKN1A* transcription [105]. In colorectal cancer cells, individual treatment with different HDACi, such as Trichostatin A, SAHA, and sodium phenylbutyrate (NaPB), strongly increased p21^cip1/waf1^ mRNA and protein levels. Specifically, the effect of NaPB, an inhibitor of predominantly nuclear HDACs, on p21^cip1/waf1^ regulation was evaluated. Treatment increased H3K27ac occupancy at the *CDKN1A* promotor and the enhancer region, demonstrating that p21^cip1/waf1^ expression is regulated by histone acetylation [114]. In pancreatic cancer cells, SAHA induced increased expression of p21^cip1/waf1^ levels as well as increased acetylation of histone H3 globally and specifically at the *CDKN1A* promotor [115]. The same was observed in multiple myeloma cells treated with the novel HDAC inhibitor Scriptaid [106]. Moreover, other studies have reported the modulation of p21^cip1/waf1^ expression after HDAC inhibition in lymphoma [116], leukemia [117], hepatocellular carcinoma [118], and breast cancer [119]; however, histone marks were not investigated.

## 4. The Role of lncRNAs and miRNAs in the Deregulation of p21^cip1/waf1^ in Cancer

**Figure 3:**
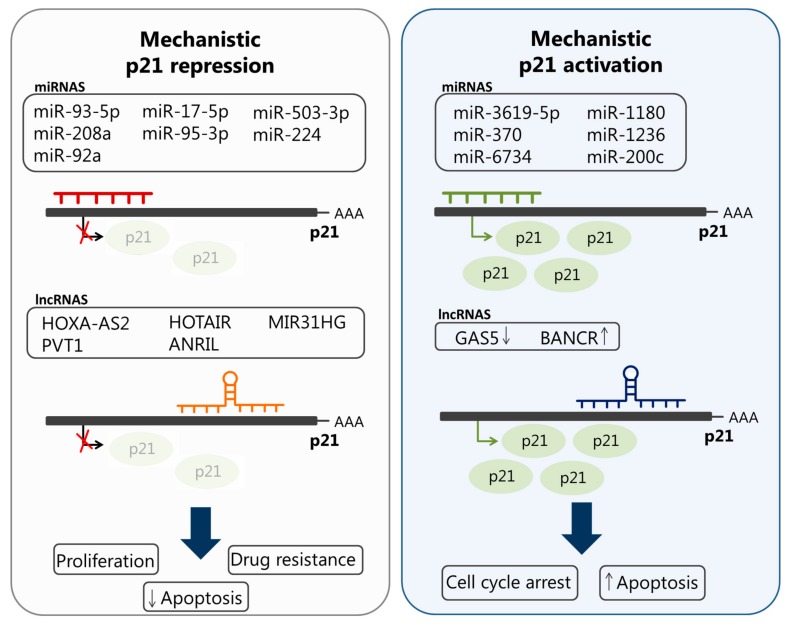
Regulation of p21^cip1/waf1^ expression by noncoding RNAs (ncRNAs). (**left figure**)—Mechanistic p21^cip1/waf1^ repression. Several microRNAs (miRNAs) and long noncoding RNAs (lncRNAs) were shown to inhibit *CDKN1A* transcription by directly interacting with its promotor. The expression of these ncRNAs in tumor cells led to p21^cip1/waf1^ repression. (**right figure**)—Mechanistic p21^cip1/waf1^ activation. On the other hand, some miRNAs were shown to enhance *CDKN1A* transcription by remodeling histones or targeting putative sites in its promotor. The expression of these miRNAs in cancer cells led to increased levels of p21^cip1/waf1^. Moreover, the BRAF-activated noncoding RNA (BANCR) interacts with p21^cip1/waf1^ to increase its expression.

Interestingly, long noncoding RNAs (lncRNA) have been shown to be involved in the regulation of p21^cip1/waf1^ (Figure 3, Table 3). Overexpression of the long noncoding RNA HOXA cluster antisense RNA2 (HOXA–AS2) is associated with tumor size, TNM stage, and a poor prognosis in gastric cancer. Interestingly, this lncRNA promotes gastric cancer cell proliferation by downregulating mRNA levels of *CDKN1A* in vitro and in vivo via binding with enhancer of zeste homolog 2 (EZH2) [102]. The long noncoding RNA plasmacytoma variant translocation 1 (PVT1) is upregulated in pancreatic duct adenocarcinoma (PDAC) tissues and has been shown to promote pancreatic cancer cell proliferation and migration, and epithelial-to-mesenchymal transition (EMT), by downregulating p21^cip1/waf1^ [120]. The HOX Antisense Intergenic RNA (HOTAIR), which is overexpressed in a wide variety of cancers, including cervical cancer, is able to silence p21^cip1/waf1^ and promote cancer radio-resistance in vitro and in vivo [121]. Other long noncoding RNAs that have been shown to silence p21^cip1/waf1^ during human malignancies include the antisense noncoding RNA in the INK4 locus (ANRIL), which is upregulated in non-small-cell lung cancer tissues. Through silencing p21^cip1/waf1^ expression, ANRIL promotes cancer cell proliferation and inhibits apoptosis. The mechanism of silencing includes binding to EZH2, in addition to a direct binding to the p21^cip1/waf1^ promotor that mediates H3K27me3 modification [122]. Recently, the lncRNA MIR31HG was found to promote cell cycle progression and proliferation in head and neck squamous cell carcinoma (HNSCC) by targeting p21^cip1/waf1^ [123]. The BRAF-activated noncoding RNA (BANCR) interacts with p21^cip1/waf1^ and increases its expression to induce cell cycle arrest and apoptosis in colorectal cancer [124].

MicroRNAs are small noncoding RNAs that are 18–25 nucleotides long [125]. They play an important role in gene regulation at the post-transcriptional level by targeting mRNAs and inducing their degradation and/or inhibiting their translation [125,126]. Notably, miRNAs can activate the expression of specific genes by targeting their promotors [127,128]. miRNAs have been strongly implicated in tumorigenesis and their deregulation is correlated with the hallmarks of cancer [129,130,131]. miR-125a and miR-125b have been shown to downregulate p53, thereby resulting in decreased expression of p21 [131,132]. Several other miRNAs have been reported to directly downregulate p21^cip1/Waf1^ by binding to its 3’-UTR, and these include miR-17, miR-20a, miR-20b, miR-93, miR-106a, and miR-106b (reviewed by Jung et al. [133]). Subsequently, upregulation of these miRNAs promotes cellular proliferation [133]. Conversely, let-7a targets Np95 ICBP90 ring finger (NIRF) and, therefore, upregulates p21 expression; however, the mechanism by which NIRF regulates p21 remains elusive [129]. Recent studies show a complicated role of miRNAs in regulating p21^cip1/waf1^ in cancer cells (Figure 1, Table 3). Plasma and tissue samples obtained from lung cancer patients showed an overexpression of miR-93-5p and a reduced expression of p21^cip1/waf1^ and Bcl-w, indicating a potential role of this miRNA in suppressing senescence through p21^cip1/waf1^ [130]. miR-208a has also been shown to be overexpressed in the serum of lung cancer patients who were subjected to radiation therapy. A further analysis indicated that this miRNA directly targets p21^cip1/waf1^ and affects the AKT/mTOR pathway to promote cell proliferation and radio-resistance. miR-208a is predicted to bind to a putative binding site at positions 86–92 in the 3’UTR of *CDKN1A* mRNA. Interestingly, this miRNA is secreted by lung cancer cells in response to radiation. Moreover, it was shown that serum miR-208a can be taken up by these cells via exosomes in a time-dependent manner [134]. A recent in vivo analysis of human cervical cancer tissue showed an upregulation of miR-92a, which is associated with cervical cancer progression. A complementary in vitro analysis revealed that p21^cip1/waf1^ is a direct target of miR-92a, which promotes cell cycle progression through inhibiting p21^cip1/waf1^ expression in cervical cancer [135]. miR-17 5p has been reported to be upregulated in multi-drug resistant human gastric cancer cell lines and its upregulation resulted in decreased expression of p21^cip1/waf1^ and drug resistance [136]. Evidence from a recent study has revealed a role of miR-95-3p in cell growth of osteosarcoma through targeting of p21^cip1/waf1^. This miRNA has been also reported to be significantly upregulated in the serum of osteosarcoma patients. The downregulation of this miRNA inhibited cell growth and induced apoptosis by enhancing the activities of caspase-3 and caspase-9 and increasing the expression of Bax/Bcl-2 proteins in osteosarcoma cells. On the other hand, the overexpression of miR-95-3p promoted cell growth, while inhibiting apoptosis, by modulating TGF-β, cyclin D1, and p21^cip1/waf1^ expression levels in these cells [137]. Similarly, miR-95-3p upregulation in a hepatocellular carcinoma (HCC) cell line, HCC tissues, and HCC xenograft mouse models was found to promote tumor proliferation and growth by directly targeting p21^cip1/waf1^. An in vitro analysis showed that the 15-bp region between 1500 bp and 1515 bp from the stop codon TAA at the 3′-UTR of *CDKN1A* contains the 3’UTR region that is targeted by this miRNA [138]. An in vitro analysis has shown that miR-503-3p induces apoptosis of lung cancer cells by downregulating p21^cip1/waf1^ and CDK4 expression [139]. miR-224 promotes chemoresistance of in vitro and in vivo models of human lung adenocarcinoma through targeting p21^cip1/waf1^ and thereby modulating G1/S transition and apoptosis [140].

On the other hand, miR-3619-5p has been shown to activate p21^cip1/waf1^ expression by targeting a putative site in its promotor. The same study showed that reduced expression of this miRNA and p21^cip1/waf1^ is associated with cancer progression and poor survival of patients with bladder carcinoma [141]. Another study showed that miR-370, miR-1180, and miR-1236 are downregulated in bladder carcinoma tissues, and their overexpression led to p21^cip1/waf1^ activation and inhibition of cancer. These miRNAs have been shown to induce the nuclear and not the cytoplasmic expression of p21 by interacting directly with its promotor. As a result, CDK4/6 and cyclin D1, which are downstream gene targets of p21^cip1/waf1^, were repressed following overexpression of the three miRNAs [142]. miR-6734 upregulates p21^cip1/waf1^ expression through modifying histones in the *CDKN1A* promoter and induces cell cycle arrest in colon cancer cells. A detailed mechanistic analysis showed that this miRNA directly interacts with the *CDKN1A* promotor by binding to a target site located at −360 to −260 relative to the transcriptional start site [143]. Results from a recent study revealed that miR-200c mediates sub-G1 and G2/M phase arrest and, consequently, enhances the radio-resistance of esophageal squamous cancer cells through modulating the expression levels of several cell cycle regulators, including p21^cip1/waf1^ [144]. The expression level of the lncRNA growth arrest specific transcript 5 (GAS5) has been observed to be downregulated in stomach cancer and resulted in decreased p21^cip1/waf1^ expression and impaired cell cycle arrest at the G1 phase. Importantly, the downregulation of GAS5 results in the downregulation of YBX1, a transactivator of p21^cip1/waf1^ in prostate cancer, thereby decreasing p21^cip1/waf1^ expression [145].

All in all, these findings show a biological significance for the role of lncRNAs and miRNAs in the deregulation of p21^cip1/waf1^ in cancer. However, the exact mechanism by which they affect the expression levels of p21^cip1/waf1^ remains unclear and requires further investigation. Nevertheless, the use of these noncoding RNAs as molecular biomarkers offers potential diagnostic and prognostic value for the better management and treatment of cancer diseases associated with deregulated p21^cip1/waf1^ expression.

**Table 3:** miRNAs and lncRNAs involved in the deregulation of p21 in cancer.

miRNA/LncRNA	Cancer Type	Expression Levels	Biological Functions	Ref.
HOXA cluster antisense RNA2 (HOXA-AS2)	GC	+	Epigenetically silences p21 transcription and promotes cancer cell growth	[102]
Plasmacytoma variant translocation 1 (PVT1)	PDAC	+	Promotes cancer cell proliferation and migration, and epithelial-to-mesenchymal transition (EMT), by downregulating p21	[120]
HOX Antisense Intergenic RNA (HOTAIR)	Cervix	+	Promotes radio-resistance by silencing p21 expression	[121]
antisense noncoding RNA in the INK4 locus (ANRIL)	NSCLC	+	Promotes cancer cell proliferation and inhibits apoptosis by silencing p21 expression	[122]
LnCRNA MIR31HG	HNSCC	+	Promotes cell cycle progression and proliferation by targeting p21	[123]
BRAF-activated noncoding RNA (BANCR)	CRC	+	Interacts with p21 and increases its expression to induce cell cycle arrest and apoptosis	[124]
miR-93-5p	Lung	+ in plasma and tissue samples obtained from lung cancer patients	Suppression of senescence through reducing the expression of p21	[130]
miR-208a	Lung	+ in serum of lung cancer patients who were subjected to radiation therapy	Reduces the expression of p21 to promote cell proliferation and radio-resistance	[134]
miR-92a	Cervix	+ in human cervical tissues and Hela cells	Promotes cell cycle progression and cell proliferation via inhibiting p21 expression	[135]
miR-17-5p	Multi-drug resistant human GC	+	Promotes drug resistance partially through downregulating p21	[136]
miR-95-3p	OS, HCC	+ in the serum of OS patients and in an HCC cell line	Promotes cell proliferation and growth by targeting p21	[137,138]
miR-503-3p	Lung cancer	+	Induces apoptosis of lung cancer cells by downregulating p21 and CDK4 expression	[139]
miR-224	Human lung adenocarcinoma	+ in cisplatin (DDP; cis-diamminedichloroplatinum II)-resistant A549 cells	Promotes chemo-resistance through targeting p21 and modulating G1/S transition and apoptosis	[140]
miR-3619-5p	BC	−	Activates p21 expression by targeting its putative promotor. Reduced expression of this miRNA and p21 is associated with cancer progression and poor survival of patients with BC	[141]
miR-370, miR-1180, and miR-1236	BC	– in BC tissues	Activates p21 and inhibits cancer	[142]
miR-6734	CC	−	Modifies histones in the p21 promoter and induces cell cycle arrest in colon cancer cells	[143]
miR-200c	ESCC	−	Mediates sub-G1 and G2/M phase arrest and, consequently, enhances radio-resistance of esophageal squamous cancer cells through modulating the expression levels of several cell cycle regulators, including p21	[144]
lncRNA growth arrest specific transcript 5 (GAS5)	GC	−	Increases p21 expression and enhances cell cycle arrest at the G1 phase	[145]

Note: Upregulation—(+); Downregulation—(–); GC—Gastric cancer; PDAC—pancreatic duct adenocarcinoma; NSCLC—non-small-cell lung cancer; HNSCC—head and neck squamous cell carcinoma; CRC—colorectal cancer; OS—osteosarcoma; HCC—hepatocellular carcinoma; BC—bladder cancer; CC—colon cancer; ESCC—esophageal squamous cell carcinoma.

## 5. Conclusions

In addition to the accumulation of multiple genetic alterations during the progression of cancer, it is now widely established that global epigenetic changes also drive carcinogenesis as sequential events. Aberrant epigenetic modifications lead to the expression or silencing of oncogenes and tumor suppressor genes, respectively. The universal cyclin-dependent kinase CDK inhibitor p21, which does not act as a transcription factor per se, is deregulated in many types of cancer. By showing dual antagonism, it is involved in many hallmarks of cancer. The expression of p21 is largely mediated by epigenetic modifications, such as DNA methylation, histone H3 methylation, and histone deacetylation. Importantly, the deregulation of miRNAs and lncRNAs contributes to p21 repression or activation, resulting in enhanced proliferation or growth arrest, drug response or resistance, and apoptosis or survival, respectively. Given that epigenetic modifications are reversible, it is conceivable that the epigenetic machinery, including noncoding RNAs, might be an important regulatory mechanism for p21 in different stages of cancer or during various therapies. The use of epigenetic drugs, in combination with conventional therapy, may lead to a novel approach for chemotherapy- and radiotherapy-resistant cancers, and alterations in p21 should not be underestimated.

## Figures and Tables

**Table 1:** Overview of p21^cip1/waf1^ regulation by epigenetic writers.

Writers	Epigenetic Mark	Experimental Conditions	p21^cip1/waf1^ Regulation	Ref.
DNMTs	5mC	Lung cancer cells	5mC affects Sp1 and Sp3 binding activity	[82]
TIP60	H2/H4ac	Decreased in colon and breast cancer	Acetylates the damage sensor ATM or the gate keeper p53	[17,18]
PCAF	H3K9/H3K14	Osteosarcoma and NSCLC cells	Expression ↓	[83]
G9a-GLP	H3K9me	Cervical cancer and acute promyelocytic leukemia cells	Recruited by Gfi1, transcription ↓	[84]
CTIP2	H3K9me3	Microglial cells	Induces transcriptional repression of p21	[85]
EZH2	H3K27me3	Erythroleukemia and NSCLC cells	Induces transcriptional repression of p21	[86,87]
DOT1L	H3K79	NSCLC	DOT1L inhibition reduces H3K79me and increases p21 expression	[88]
PRMT6	H3R2me2	Prostate cancer	PRMT6 silencing leads to increased p21 expression	[89]
Suv4-20h1	H4K20me	Myeloid leukemia cells	Induces transcriptional repression of p21	[90]
	H2A.Z	Osteosarcoma cells	Reduces binding of TIP60 to p21 promotor and activates p21 transcription	[91,92]

DNMTs—DNA methyltransferases; EZH2—enhancer of zeste homolog 2; G9a-GLP—G9a and G9a-like protein; CTIP2—chicken ovalbumin upstream promoter transcription factor-interacting protein 2; DOT1L—disruptor of telomeric silencing 1-like; PRMT6—protein arginine-N-methyltransferase; Suv4-20h1—suppressor of variegation 4-20 homolog 1; H2—histone H2; H4ac—histone H4 acetylation; H3K9/H3K14—histone H3 lysine 9/histone H3 lysine 14; H3K9me—histone H3 lysine 9 methylation; H3K9me3—histone H3 lysine 9 trimethylation; H3K79—histone H3 lysine 79; H3R2me2—histone H3 arginine 2 dimethylation; H4K20me—histone H4 lysine 20 methylation; H2A.Z—histone H2 A variant Z; NSCLC—non-small cell lung cancer; ATM—ataxia telangiectasia mutated.

**Table 2:** Overview of p21^cip1/waf1^ regulation by epigenetic erasers.

Erasers	Epigenetic Mark	Experimental Conditions	p21^cip1/waf1^ Regulation	Ref.
TET	5hmC	Glioma cells	Expression ↑	[104]
KDM1A/LSD1	H3K4/H3K9me	Renal cells carcinoma and glioma	Regulates p21^cip1/waf1^ via demethylation of histone and non-histone targets	[100,101]
HDAC	Histone deacetylation	Multiple myeloma and NSCLC	Expression ↑	[105,106]

TET—ten-eleven translocation; KDM1A/LSD1—lysine-specific (histone) demethylase 1; HDAC—histone deacetylases; 5hmC—5-hydroxymethylcytosine; H3K4/H3K9me—histone H3 lysine 4/histone H3 lysine 9 methylation; NSCLC—non-small cell lung cancer.
